# A Comparison of Dobutamine, Norepinephrine, Vasopressin, and Hetastarch for the Treatment of Isoflurane-Induced Hypotension in Healthy, Normovolemic Dogs

**DOI:** 10.3390/ani13162674

**Published:** 2023-08-19

**Authors:** Natalia Henao-Guerrero, Carolina H. Ricco-Pereira, Vaidehi V. Paranjape

**Affiliations:** 1 Department of Small Animal Clinical Sciences, Virginia-Maryland College of Veterinary Medicine, Blacksburg, VA 24061, USA; nguerrer@vt.edu; 2 Department of Veterinary Clinical Sciences, The Ohio State University-College of Veterinary Medicine, Columbus, OH 43210, USA; riccopereira.1@osu.edu

**Keywords:** canine, inhalants, volatile anesthetics, pulmonary artery thermodilution, inotrope, vasopressor, colloids, arterial blood pressure, hemodynamics, general anesthesia

## Abstract

**Simple Summary:**

In anesthetized dogs, hypotension, defined as a mean arterial pressure (MAP) of <60–70 mmHg, has an incidence of up to 60%. It is therefore imperative to avoid the occurrence of hypotension and undertake immediate strategies to treat it. Treatment protocols for the correction of isoflurane-mediated hypotension include lowering or terminating isoflurane administration; lowering isoflurane requirement by using anesthetic adjuncts and analgesics; intravenous crystalloid and/or colloid bolus; and delivering anticholinergics, positive inotropes, and vasopressor drugs. The present study evaluated and compared the hemodynamic effects of dobutamine (DOB), norepinephrine (NEP), vasopressin (VAS), and hetastarch (HES) for the treatment of isoflurane-induced hypotension and established the most effective dosage regimen for the correction of hypotension. In healthy, anesthetized Beagle dogs undergoing acute, severe isoflurane-induced hypotension, NEP administered as an infusion was the most efficient therapy for correcting MAP. However, the DOB, VAS, and HES treatments were unsuccessful in stabilizing the MAP to >65 mmHg, even at maximum doses.

**Abstract:**

Isoflurane is a commonly used inhalation anesthetic in species undergoing veterinary care that induces hypotension, impacting organ perfusion, making it imperative to minimize its occurrence or identify effective strategies for treating it. This study evaluated and compared the hemodynamic effects of DOB, NEP, VAS, and HES in twelve isoflurane-anesthetized Beagle dogs. The order of the first three treatments was randomized. HES was administered last. Data were collected before treatments (baseline) and after 10 min of a sustained MAP of <45 mmHg induced by a high end-tidal isoflurane concentration (T0). Once treatment was initiated and the target MAP was achieved (65 to 80 mmHg) or the maximum dose reached, data were collected after 15 min of stabilization (T1) and 15 min after (T2). A 15 min washout period with a MAP of ≥65 mmHg was allowed between treatments. The intravenous dosage regimens started and were increased by 50% every five minutes until the target MAP or maximum dose was reached. The dosages were as follows: DOB, 5–15 μg/kg/min; NEP, 0.1–2 μg/kg/min; VAS, 0.5–5 mU/kg/min; and HET, 6% 1–20 mL/kg/min. DOB improved CO, DO_2_, and VO_2_, but reduced SVR. VAS elevated SVR, but decreased CO, DO_2_, and VO_2_. HES minimally changed BP and mildly augmented CO, DO_2_, and VO_2_. These treatments failed to reach the target MAP. NEP increased the arterial BP, CO, MPAP, and PAWP, but reduced HR. Norepinephrine infusion at 0.44 ± 0.19 μg/kg/min was the most efficient therapy for correcting isoflurane-induced hypotension.

## 1. Introduction

Volatile anesthetics are an integral component of general anesthesia in research settings and clinical practice in veterinary and human medicine. Throughout the past several decades, a large number of studies that have defined the cardiac, vascular, and autonomic effects of volatile anesthetics have been published. These effects become evident immediately following anesthetic induction and during anesthetic maintenance. Some of the key elements of the cardiovascular system that are impacted include: (i) myocardial contractility, (ii) systemic vascular resistance (SVR), and (iii) cardiac electrophysiology [1].

The dose-dependent depression of myocardial contractility is primarily due to the dysregulation of the sarcolemmal L-type Ca^2+^ channels, sarcoplasmic reticulum, and contractile proteins [2,3]. Experimental canine studies have revealed that there are differences between inhalant anesthetics in this regard and that halothane and enflurane exert equal but more potent negative inotropic effects than isoflurane, desflurane, or sevoflurane [4,5]. Halothane exhibits the most pronounced reduction in blood pressure (BP) via myocardial depression and decrease in arterial tone, while isoflurane, sevoflurane, and desflurane primarily alter the left ventricular afterload and SVR [2,6,7,8]. Peripheral vasodilation and hypotension worsen with increasing concentrations of volatile anesthetics in a dose-dependent fashion. The association between intraoperative hypotension and its duration with postoperative mortality and organ dysfunction after general anesthesia is well documented [9]. All volatile agents attenuate the baroreceptor reflex, where every constituent of the reflex arc (i.e., afferent and efferent activity, central processing) can be inhibited [10,11]. Volatile anesthetics also slow down the rate of sinoatrial node discharge by affecting the sinoatrial node automaticity, along with having direct action on cardiac pacemaker cells and conduction pathways [2,12]. Canine studies have suggested that the myocardial sensitization to arrythmias is halothane > enflurane > sevoflurane = isoflurane [13,14].

Isoflurane is a commonly used inhalation anesthetic in animal species under veterinary care. Just like other volatile anesthetics, isoflurane anesthesia induces hypotension and consequently results in a decline in organ perfusion and poor patient outcomes [15]. In anesthetized dogs, hypotension, defined as a mean arterial pressure (MAP) of <60–70 mmHg [16,17], has an incidence of up to 60%, which even includes elective surgeries [18,19]. It is therefore imperative to avoid the occurrence of hypotension and undertake immediate strategies to treat it. Treatment protocols for the correction of isoflurane-mediated hypotension include lowering or terminating isoflurane administration; lowering isoflurane requirement by using anesthetic adjuncts and analgesics, intravenous crystalloid and/or colloid bolus; and delivering anticholinergics, positive inotropes, and vasopressor drugs [16,20,21]. Dobutamine exhibits a positive inotropy via β_1_ adrenergic agonist activity, thus increasing cardiac output (CO) in a dose-dependent fashion. Another routine treatment for hypotension is norepinephrine, which has potent agonistic actions on the α_1_ and α_2_ adrenergic receptors, resulting in peripheral vasoconstriction and venoconstriction, along with well-established β_1_ agonism-driven cardiac stimulation [16,20]. Vasopressin is also used for its vasoconstrictor effects, through its V_1a_ receptor action. In the face of acidemia, it can enhance the SVR and can be useful in treating refractory hypotension, as seen in sepsis or hypovolemic shock [20,21]. These drugs are commonly selected for maintaining the BP in hypotensive and critically ill small animals. Synthetic colloidal solutions are popular in veterinary medicine and can be effective plasma volume expanders, administered alone or following a crystalloid or hypertonic saline bolus [22,23].

Even though these therapeutic options are well described in the literature [20,21,22,23], there are limited studies specifically focused on which of these therapies is most effective against severe isoflurane-induced hypotension in anesthetized dogs. Moreover, the dosages of dobutamine, norepinephrine, and vasopressin required to successfully combat and correct severe hypotension from isoflurane are relatively unknown. The objectives of the present study concerning healthy, normovolemic anesthetized dogs were: (i) to evaluate and compare the hemodynamic effects of dobutamine, norepinephrine, vasopressin, and hetastarch for the treatment of isoflurane-induced hypotension and (ii) to establish the most effective dosage regimen for dobutamine, norepinephrine, and vasopressin during the correction of hypotension. We hypothesized that isoflurane-induced hypotension would be similarly and successfully treated with dobutamine, norepinephrine, and vasopressin, but not with hetastarch.

## 2. Materials and Methods

### 2.1. Animals

Twelve adult, healthy, female, purpose-bred, and university-owned Beagles aged 12–24 months, with body weights of 8.5 ± 1.2 kg, participated in this prospective, crossover, randomized experimental study. The dogs were certified as ‘healthy’ based on a thorough physical examination, cardio-pulmonary auscultation, heart worm test, complete blood count, and serum chemistry. The experimental study and use of animals were approved by the Virginia Tech University Institutional Animal Care and Use Committee (protocol number 12-094-CVM). Six weeks after the end of the study, all the dogs underwent surgical sterilization, vaccination, and deworming, and were successfully adopted into single-family homes.

Based on previous canine studies evaluating the effect of an intervention on CO [24,25], a sample size of a minimum of ten animals was required to demonstrate a 25% significant difference in CO in response to the treatment administered, assuming a statistical power of 0.8 and an alpha level of 0.05 (http://estatistica.bauru.usp.br/calculoamostral/; accessed on 23 August 2012). An additional two dogs were added to the sample size to improve the statistical power of the study, as well as to account for unforeseen circumstances (e.g., missing data or challenging instrumentation).

### 2.2. General Anesthesia and Anesthetic Monitoring

After the completion of a two-week acclimatization period, each dog was randomly selected for the experiment, and food was withheld for 12 h with ad libitum access to water. On the day of the experiment, a cephalic catheter was aseptically placed, followed by anesthetic induction via isoflurane delivery in 100% oxygen (4 L/min) using a fitted facemask connected to a small animal anesthesia machine via a Bain non-rebreathing system. Once the appropriate anesthetic plane and adequate muscle relaxation for intubation were confirmed, the trachea was intubated, and a cuffed endotracheal tube was secured to the dog with a tie gauze and connected to the non-rebreathing circuit and anesthesia machine (Apollo; Draeger Medical Inc., Telford, PA, USA). The dogs were then placed in dorsal recumbency for the entire anesthetic period. Isoflurane in 100% oxygen (1–2 L/min) was used for anesthetic maintenance. The end-tidal concentration of isoflurane (ET_ISO_), as continuously measured using an infrared gas analyzer, was included in a multiparameter monitor (Datex-Ohmeda S/5 Compact anesthesia monitor; GE Healthcare, Chicago, IL, USA) and was targeted at 1.4–1.6%, except during the experimental procedures, which required increments in ET_ISO_. The other standard anesthetic monitoring included in the same monitor were a lead II electrocardiogram for heart rate (HR) and rhythm, an end-tidal concentration of carbon dioxide, esophageal temperature, a peripheral oxygen saturation of hemoglobin (SpO_2_), and spirometry. Normothermia (36.9 to 37.8 °C) was maintained in all dogs throughout the anesthetic period with a forced-air warming device and circulating water blanket in place. Standard monitoring variables were recorded every five minutes as per routine standards throughout the anesthetic period. No maintenance fluids were administered.

The dogs were mechanically ventilated using volume-controlled ventilation settings with a tidal volume of 10–12 mL/kg, and the respiratory rate was adjusted to maintain the end-tidal carbon dioxide concentration between 35 and 45 mmHg. Mechanical ventilation was initiated to maintain the consistent values in a specified range for the end-tidal carbon dioxide and isoflurane concentrations throughout the study. Aseptic arterial catheterization in the dorsal pedal artery was performed for recording invasive systolic (SAP), diastolic (DAP), and mean arterial blood pressure (MAP), as well as periodic arterial blood gas sampling. The same multi-parametric monitor was connected to a heparinized, saline-flushed (3 IU/mL) disposable pressure transducer system (Deltran II; Utah Medical Products Inc., Midvale, UT, USA), which was zeroed and leveled approximately at the location of the right atrium. Prior to the beginning of the experiment, the transducer and monitor were calibrated against a digital pressure manometer. With the help of a 2-point calibration technique (0 and 150 mmHg), the accuracy and linearity of the transducer were assessed via verification against the mercury manometer. The calibration was deemed to be acceptable when the difference between the manometer and displayed monitor pressures was <2 mmHg.

### 2.3. Instrumentation for Intermittent Pulmonary Artery Thermodilution to Measure CO and Other Hemodynamic Variables

By performing a modified Seldinger technique, a 6 Fr 8.5 cm hemostasis introducer (Fast-Cath; Abbott Cardiovascular, Plymouth, MN, USA) was aseptically inserted into the right jugular vein, through which a 5 Fr 75 cm Swan Ganz thermodilution catheter (132FS; Edwards Lifesciences Corp., Irvine, CA, USA) was advanced. The Swan Ganz catheter was maneuvered based on the pressure–waveform relationship displayed on the CO monitor (Carescape B850; GE Healthcare, Chicago, IL, USA). This was continued until the distal tip was located in the pulmonary artery. A 3 mL, 2 °C to 5 °C cold saline bolus was used as an indicator and injected into the proximal injectate port for the CO determination. The other hemodynamic variables measured using this catheter were the central venous pressure (CVP; via proximal port), mean pulmonary artery pressure (MPAP; via distal port), and pulmonary artery wedge pressure (PAWP; via distal port and balloon inflation). A detailed explanation of the thermodilution procedure in dogs was published previously and is available for review [26,27,28]. Each CO reading was timed at the end of the expiration, and the saline bolus was injected for over more than three seconds. At each datapoint, the CO reading recorded equaled the mean of 3 consecutive measurements within a 10% variation. This thermistor-tipped Swan Ganz catheter was also used to measure the core body temperature.

### 2.4. Calculations for Other Hemodynamic Variables Derived from the Thermodilution Catheter

Various other hemodynamic variables, such as the systemic vascular resistance (SVR), pulmonary vascular resistance (PVR), arterial oxygen content (CaO_2_), venous oxygen content (CvO_2_), oxygen delivery (DO_2_), oxygen consumption (VO_2_), and oxygen extraction ratio (OER), were calculated using the following formulae [19,27,29]:

$$\mathrm{SVR}\;(\mathrm{dyn}\cdot \sec/{\mathrm{cm}}^{5})=\;\frac{(\mathrm{Mean}\;\mathrm{Arterial}\;\mathrm{Pressure}\;-\mathrm{Central}\;\mathrm{Venous}\;\mathrm{Pressure})\;\times \;80}{\mathrm{Cardiac}\;\mathrm{output}\;\mathrm{by}\;\mathrm{Thermodilution}}$$

$$\mathrm{PVR}\;(\mathrm{dyn}\cdot \sec/{\mathrm{cm}}^{5})=\;\frac{(\mathrm{Mean}\;\mathrm{Pulmonary}\;\mathrm{Arterial}\;\mathrm{Pressure}\;-\mathrm{Pulmonary}\;\mathrm{Artery}\;\mathrm{Wedge}\;\mathrm{Pressure})\;\times \;80}{\mathrm{Cardiac}\;\mathrm{output}\;\mathrm{by}\;\mathrm{Thermodilution}}$$

CaO_2_ (mL/dL) = (1.34 × SaO_2_ × Hb) + (0.003 × PaO_2_)

CvO_2_ (mL/dL) = (1.34 × SvO_2_ × Hb) + (0.003 × PvO_2_)

DO_2_ (mL/min) = 10 × CaO_2_ × CO

VO_2_ (mL/min) = CO × (CaO_2_ − CvO_2_)

OER (%) = (CaO_2_ − CvO_2_)/CaO_2_ × 100

where SaO_2_ = arterial oxygen saturation; SvO_2_ = venous oxygen saturation; Hb = hemoglobin concentration; PaO_2_ = arterial oxygen tension; and PvO_2_ = venous oxygen tension.

### 2.5. Administration for Treatments and Data Collection

**Figure 1:**
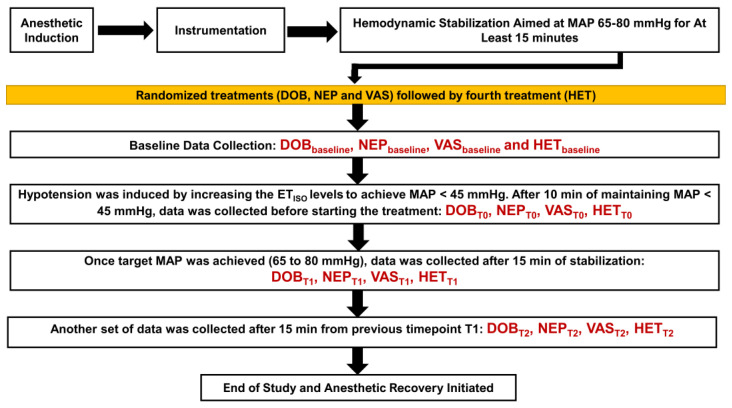
Study timeline and data collection from twelve isoflurane-anesthetized Beagle dogs subjected to four treatments. Three treatments, i.e., dobutamine (DOB), norepinephrine (NEP), and vasopressin (VAS) were always administered in a randomized order. The colloid treatment (HET) was always administered last. Baseline data were collected before DOB (DOB_baseline_), NEP (NEP_baseline_), VAS (VAS_baseline_), and hetastarch (HET_baseline_) treatments. Immediately after this, hypotension was induced by increasing the end-tidal isoflurane concentration to achieve mean arterial pressure (MAP) < 45 mmHg. After 10 min of maintaining MAP < 45 mmHg, data were collected before starting treatment (i.e., DOB_T0_, NEP_T0_, VAS_T0_, and HET_T0_). The intravenous dosage regimens for the four treatments were: (i) DOB—starting dose of 5 μg/kg/min, which was increased by 50% every five minutes until MAP was 65 to 80 mmHg or maximum dose of 15 μg/kg/min; (ii) NEP—starting dose of 0.1 μg/kg/min, which was increased by 50% every five minutes until MAP was 65 to 80 mmHg or maximum dose of 2 μg/kg/min; (iii) VAS—starting dose of 0.5 mU/kg/min, which was increased by 50% every five minutes until MAP was 65 to 80 mmHg or maximum dose of 5 mU/kg/min; and (iv) HET—6% 670/0.75 hetastarch administered at 1 mL/kg/min until MAP was 65 to 80 mmHg or maximum dose of 20 mL/kg. Once the treatment was initiated and target MAP was achieved (65 to 80 mmHg) or maximum dose was reached, data were collected after 15 min of stabilization (i.e., DOB_T1_, NEP_T1_, VAS_T1_, and HET_T1_) and 15 min after timepoint T1 (i.e., DOB_T2_, NEP_T2_, VAS_T2_, and HET_T2_). There was a minimum 15 min washout period between treatments where MAP values were ≥65 mmHg.

Once the instrumentation was completed, hemodynamic stabilization was achieved by aiming for an MAP of 65 to 80 mmHg for at least 15 min. Then, each dog underwent four treatments (Figure 1). Three treatments, i.e., dobutamine (DOB), norepinephrine (NEP), and vasopressin (VAS), were always administered in a randomized order using an online website module (https://www.randomizer.org/; accessed on 25 August 2012). However, the colloid treatment (HET) always followed after the other three treatments were administered. Baseline data were collected before the DOB (DOB_baseline_), NEP (NEP_baseline_), VAS (VAS_baseline_), and 6% 670/0.75 hetastarch (HET_baseline_) treatments. After collecting the baseline data for the selected treatment, hypotension was induced by increasing the ET_ISO_ levels to achieve an MAP of <45 mmHg. After 10 min of maintaining an MAP of <45 mmHg, data were collected before starting treatment (i.e., DOB_T0_, NEP_T0_, VAS_T0_, and HET_T0_). The dosage regimens for the four treatments were: (i) DOB—intravenous dobutamine via an infusion pump (BD Alaris™ Infusion System; Becton, Dickinson and Company, Franklin Lakes, NJ, USA) was administered at a starting dose of 5 μg/kg/min, and the dose was increased by 50% every five minutes until the MAP was 65 to 80 mmHg; (ii) NEP—intravenous norepinephrine via an infusion pump (BD Alaris™ Infusion System) was administered at a starting dose of 0.1 μg/kg/min, and the dose was increased by 50% every five minutes until the MAP was 65 to 80 mmHg; (iii) VAS—intravenous vasopressin via an infusion pump (BD Alaris™ Infusion System) was administered at a starting dose of 0.5 mU/kg/min, and the dose was increased by 50% every five minutes until the MAP was 65 to 80 mmHg; and (iv) HET—intravenous 6% 670/0.75 hetastarch via an infusion pump (BD Alaris™ Infusion System) was administered at 1 mL/kg/min until the MAP was 65 to 80 mmHg. The maximum allowable limit for the dosages were 15 μg/kg/min (DOB), 2 μg/kg/min (NEP), 5 mU/kg/min (VAS), and 20 mL/kg (HET). Once the treatment was initiated and the target MAP was achieved (65 to 80 mmHg) or the maximum dosage was reached, data were collected after 15 min of stabilization (i.e., DOB_T1_, NEP_T1_, VAS_T1_, and HET_T1_), followed by another data collection, which was 15 min after timepoint T1 (i.e., DOB_T2_, NEP_T2_, VAS_T2_, and HET_T2_). At least a 15 min washout period was assigned between the treatments, and it was ensured that the MAP values were ≥65 mmHg during the rest/washout period. All the hemodynamic data were acquired during the expiratory phase of the respiratory cycle. An arterial and venous blood gas analysis was performed at baseline and timepoints T0 and T2.

### 2.6. Anesthetic Recovery

After the HET data collection, the jugular and arterial catheters were removed and external pressure was applied to confirm that bleeding and hematoma were absent. The delivery of isoflurane was terminated. After extubation, 0.2 mg/kg of IV butorphanol was administered to all the dogs and they were moved to individual kennels. A blood sample was obtained after 24 h to confirm that the hematocrit, total protein, and blood urea nitrogen levels were normal. The cardiorespiratory parameters, pain assessment and catheter sites were monitored periodically every 4–8 h for the next 48 h.

### 2.7. Statistical Analysis

The outcomes included ET_ISO_, SpO_2_, HR, SAP, MAP, DAP, CO, CVP, MPAP, PAWP, SVR, PVR, DO_2_, VO_2_, and OER. Normal probability plots and a Shapiro–Wilk test showed that all the outcomes followed a normal distribution. Subsequently, the data were summarized as mean ± standard deviation. The effects of the treatment (DOB, NEP, VAS, and HET) and time period (baseline, T0, T1, and T2) on each outcome were assessed using a mixed-model analysis of variance. The generalized linear model specified treatment, time, and the interaction between treatment and time as fixed effects. Dog identification was the random effect, and a Kenward–Roger approximation was the degrees of freedom denominator. The interaction between treatment and time was further analyzed to compare the treatments at each time point and compare the time points within each treatment. The overall *p*-values for the main effects (treatment and time) across all the outcomes were adjusted for multiple testing using the Benjamini–Hochberg false discovery rate method. For each outcome with a significant overall *p*-value (after the Benjamini–Hochberg adjustment), *p*-values for 2-way comparisons were adjusted for multiple comparisons using Tukey’s procedure. Significance was set at *p* < 0.05. All the analyses were performed using SAS version 9.3 (Cary, NC, USA).

## 3. Results

All the dogs exhibited smooth and uneventful anesthetic induction, maintenance, and recovery. The successful placement of a Swan Ganz catheter was performed in all the dogs without any complications arising from the right heart catheterization. Even though all twelve dogs successfully underwent the experiment, significant missing data were reported in two dogs under the HET and DOB treatments. This made a data analysis challenging, and these two dogs were thus eliminated from the study and their data were not included. The dogs were normothermic (37.3 ± 0.5 °C) and normocapnic (40 ± 4 mmHg) throughout the anesthetic period. The jugular and arterial catheter sites did not present any evidence of hematoma formation or subcutaneous bruising. Four out of the twelve dogs required an additional dose of 0.3 mg/kg of IV butorphanol due to a display of mild pain at the 4 h timepoint post-extubation based on a pain assessment. The cardiorespiratory parameters, appetite, demeanor, and excretory functions were within the normal limits throughout the 48 h post-extubation period. The blood samples obtained at 24 h showed the hematocrit, total protein, and blood urea nitrogen levels to be within normal limits for all the dogs. Between the ten dogs, there were no differences in the: (i) total anesthesia time (*p* = 0.94); (ii) time spent between anesthetic induction to recording DOB_baseline_ (*p* = 0.43), NEP_baseline_ (*p* = 0.89), VAS_baseline_ (*p* = 0.21), and HET_baseline_ (*p* = 0.55); (iii) time spent between the baseline and T2 data collection (*p* = 0.41); and (iv) time spent obtaining data for timepoints T1 (*p* = 0.67) and T2 (*p* = 0.14) during the treatments. The final doses required to achieve the target MAP values (65 to 80 mmHg) during isoflurane-induced hypotension were 9.64 ± 4.33 μg/kg/min for dobutamine infusion, 0.44 ± 0.19 μg/kg/min for norepinephrine infusion, 5 ± 0 mU/kg/min for vasopressin infusion, and 20 ± 0 mL/kg for hetastarch. Additionally, there was no change in the parameters recorded from the arterial and venous blood gas analysis performed at baseline or timepoints T0 and T2.

There were no differences between the baseline data for the measured parameters during the four treatments, as shown in Table 1. The ET_ISO_ values that caused an MAP of <45 mmHg in the four treatment groups were not different from one another. Once the hypotension set point from isoflurane was established, the dobutamine infusion caused mild increments in the heart rate, especially at high doses, with significant increases in the arterial BP, CO, MPAP, and PAWP values at T1 and T2 as compared to T0 (*p* < 0.001); however, the CVP values were significantly higher only at T2 compared to baseline (*p* < 0.001). Although the MAP values were increased as compared to hypotension, a consistent target MAP (65 to 80 mmHg) was not achieved with the doses used in the study. The norepinephrine infusion significantly decreased HR at T1 and T2 as compared to baseline and T0, with a subsequent increase in arterial BP, CO, MPAP, and PAWP as compared to T0 (*p* < 0.001). However, CO was significantly lower at T1 (*p* = 0.033) and T2 (*p* = 0.026) versus at baseline. Additionally, there was a small increase in CVP at T1 (*p* = 0.015) and T2 (*p* = 0.037) as compared to baseline. The administration of vasopressin also caused a significantly lower HR at T1 and T2 as compared to baseline and T0, with a concurrent increase in arterial BP, MPAP, and PAWP in comparison to T0 (*p* < 0.001). Even with the highest dose limit, a consistent target MAP (65 to 80 mmHg) was not achieved. The CO values were observed to deteriorate further with vasopressin, while no change in CVP was seen—as opposed to at T0. During the hetastarch treatment, HR was significantly elevated at T1 and T2 as compared to baseline and T0, but hypotension was not corrected. CO mildly increased with a significant rise in CVP, MPAP, and PAWP at T1 and T2 as compared to T0.

Similar to the measured parameters, there were no differences between the baseline data for the calculated parameters during the four treatments, as shown in Table 2. Dobutamine significantly decreased the SVR at T1 and T2 as compared to baseline and T0, while lowering the PVR at T1 and T2 as compared to T0 (*p* < 0.001). Meanwhile, norepinephrine and vasopressin caused significant increments in the SVR and PVR at T1 and T2 as compared to baseline (*p* < 0.001). The administration of hetastarch significantly lowered the SVR with concurrent elevations in the PVR at T2 as compared to baseline and T0 (*p* < 0.001). The dobutamine infusion led to significant increases in DO_2_ and VO_2_ at T1 and T2 as compared to T0 (*p* < 0.001); however, these values had no difference from baseline. During severe hypotension, norepinephrine significantly improved DO_2_ and VO_2_ at T1 and T2; however, these values were significantly lower than at baseline (*p* < 0.001). Vasopressin significantly diminished DO_2_ and VO_2_ at T1 and T2 as compared to baseline and T0 (*p* < 0.001). Hetastarch augmented DO_2_ and VO_2_ when the MAP was <45 mmHg. The OER did not show any significant changes throughout the experiment.

## 4. Discussion

The pathways that control arterial BP depend upon the length of period during which abnormal fluctuations in BP occur, i.e., short- and long-term effects. When acute variations in BP occur over minutes to hours, short-term control regulates BP through changes impacting CO, SVR, arterial compliance, heart rate, blood volume, and myocardial contractility [30]. Autoregulation ensures that the optimum blood flow and oxygen delivery are provided to the critical organs by keeping the blood pressure values within a physiologic range. The main elements of the control system that aims to stabilize adequate perfusion are the baroreceptors, chemoreceptors, central pressure centers in the medulla oblongata, and the renin–angiotensin–aldosterone pathway. Volatile anesthetics can alter autoregulatory mechanisms and, therefore, the vigilant monitoring and treatment of blood pressure can be a crucial step in maintaining a steady hemodynamic status under general anesthesia. Hypotension, i.e., an MAP of <60–70 mmHg under general anesthesia, can be triggered by decreases in HR, CO, stroke volume, and SVR [15,16]. Balanced anesthesia techniques incorporating analgesic infusions and regional anesthesia permit a decrease in the requirement for the inhalant anesthetic in order to diminish its vasodilatory impact, while other treatment strategies revolve around (i) the normalization of venous return and CO with intravenous fluid therapy using crystalloids, hypertonic saline, and colloids; (ii) the manipulation of vasomotor tone with sympathomimetic vasopressors such as norepinephrine and non-adrenergic vasopressors such as vasopressin; (iii) improving myocardial contractility with specific inotropes such as dobutamine; and (iv) the treatment of bradycardia with anticholinergics. Addressing other causative factors (i.e., arrhythmias, hypoproteinemia, hypoglycemia, and electrolyte imbalances) can also be beneficial in correcting BP values [16]. In the present study, we selected pharmacological interventions based on their mechanism of action, hemodynamic variable targeted (i.e., venous return, CO, SVR, and inotropy), and outcome on BP values. We found the norepinephrine infusion to be the most effective intervention during the treatment of isoflurane-induced hypotension (MAP < 45 mmHg) at 0.44 ± 0.19 μg/kg/min.

The detrimental effects of inhalant anesthetics can be centered on inotropy, vasomotor tone, and electrophysiologic function. The reduction in calcium ion flux through sarcolemmal L-type Ca^2+^ channels diminishes the availability of intracellular Ca^2+^ that can bind to the troponin–actin–myosin complex, which is responsible for myocardial contraction. Another mechanism directly activates the ryanodine-sensitive sarcoplasmic reticulum Ca^2+^ channels, thus negatively affecting Ca^2+^ storage and release during systole [2,3]. The severity of these changes may be greater in a preexistent depressed myocardium than in a normal myocardium. If contractility declines, it further lowers the ventricular emptying during systole, thus increasing the end-systolic volume and decreasing the stroke volume. The end result of this cascade is diminished CO and a subsequent reduction in blood pressure. A similar phenomenon occurs at the level of arterioles, where a decline in Ca^2+^ ion influx in the smooth muscles results in a diminished resting tone and, consequently, a reduced SVR and BP. Endothelial-dependent mechanisms include an effect on bradykinin levels, the activation of endothelial nitric oxide synthase, the release of nitric oxide, endothelin-1 production, angiotensin II-induced vascular smooth muscle contraction, Ca^2+^ ion sensitivity, and a calcium-induced calcium release process [2]. Halothane and enflurane are more potent at impairing the baroreceptor reflex arc than isoflurane, sevoflurane, and desflurane. Older inhalant anesthetics such as halothane may not appreciably change the heart rate in humans as opposed to isoflurane or desflurane, which increase the heart rate in response to a decrease in BP due to the preservation of the baroreceptor reflex [10,11]. Outcomes include the shortening of the cardiac action potential and effective refractory period duration in normal Purkinje fibers, prolonged ventricular conduction times, a delayed atrioventricular conduction time, and refractoriness [2,12]. Hence, these agents can cause bradycardia or bradyarrhythmias, which, if untreated, can contribute to hypotension. The molecular mechanisms underlying a reduction in the arrhythmogenic threshold for epinephrine exhibited by the inhalant anesthetics are poorly understood. The proposed theory includes the modulation of L-type Ca^2+^ ion channels that shorten the refractory period or an inhibitory action on voltage-gated K^+^ channels that may disturb repolarization and increase the risk of ventricular arrhythmias [13].

Isotonic crystalloids have been extensively evaluated for fluid resuscitation in hemodynamically unstable patients and are often the first choice during sepsis to normalize the MAP, lactate levels, and central venous oxygen saturation. Interestingly, it has been shown that the intravenous administration of isotonic fluid therapy at 15 to 80 mL/kg per hour during normotension and hypotension does not increase the BP in anesthetized normovolemic canine patients [31,32,33]. If hypotension in anesthetized canines was an outcome of a deep plane of anesthesia, administering isotonic fluids at 1 mL/kg/min did not increase CO or stroke volume. In recovery, vomiting, facial pitting edema, and nasal discharge were observed in some of the dogs due to the high-volume crystalloids administered [34]. Diarrhea, respiratory distress, and protrusion of the ocular globe have also been reported [31]. When an isotonic crystalloid or a colloid was infused intravenously during isoflurane-induced hypotension, lactated Ringer’s solution did not increase SAP, but decreased blood viscosity, packed cell volume, and total protein concentration, while hetastarch increased colloid osmotic pressure. Both types of fluids increased CO and blood volume, but decreased SVR. The volume of lactated Ringer’s solution to restore SAP was higher and this change in BP was achieved approximately three times slower in anesthetized dogs with blood loss-associated hypotension [25]. These canine studies caution against the use of crystalloid fluids to counteract hypotension under general anesthesia in normovolemic patients [31,32,33,34].

In numerous studies with anesthetized dogs, hetastarch has been proven to be beneficial over a crystalloid solution in normalizing hemodynamics during hypovolemic shock and hypotension [25,31,35,36]. In our study, we selected hetastarch for its superior effects on volume expansion, the longer duration of its effect, and the optimization of plasma colloid osmotic pressure. We observed increases in HR, CO, CVP, MPAP, PAWP, DO_2_, and VO_2_, with minimal to no change in arterial BP. It is possible that, in anesthetized, hypotensive, normovolemic patients, such as our study dogs, colloids have a lower impact on BP since the infused volume also lowers the SVR. In this scenario, Ohm’s law, i.e., BP = CO × SVR, explains why the BP remains stable as the colloid infusion causes an expansion of the blood and plasma volume. The effect of the volume expansion can also increase inotropy, which further augments the stroke volume and CO. The administration of colloids can lower the blood viscosity via the hemodilution of the packed cell volume and total protein [37], which, in correspondence with a reduced SVR, will ease cardiac workload and functioning [23,31,34]. We attribute the rise in HR in response to the hetastarch infusion to the Bainbridge or atrial reflex, where the acute increase in blood volume improved the venous return to the heart, thus accelerating the HR via a reduced parasympathetic tone via the efferent vagal pathway, triggered by the stimulation of atrial stretch receptors [38]. The current evidence demonstrates that there may be a lower risk for hetastarch-associated acute kidney injury in small animals when small bolus doses (<20 mL/kg) are used for a short duration. Having said that, its use in pre-existing azotemia is not recommended [23]. Colloid use in critically ill and septic patients might be risky due to the possible damage to the endothelial glycocalyx that can exacerbate the extravasation of fluids. Moreover, the negative effects of hetastarch in dogs, with or without preexisting coagulopathy, can be observed through coagulation tests even at doses of <20 mL/kg [39]. In a retrospective study on the intra-anesthetic predictors of prolonged hospitalization and survival for canine patients at a veterinary teaching hospital, colloid use had a significant impact [40]. Hence, clinicians must exercise caution and weigh the benefits against the risks associated with colloid therapy, along with a continuous assessment of the hemodynamic status in anesthetized canine patients.

Dobutamine, when used clinically, is a racemic mixture, where the pharmacodynamic activity depends on the interactions of the individual properties of the (+) and (−) stereoisomers. The (−)-dobutamine is a potent adrenergic α_1_ agonist with weak β_1_ and β_2_ activity effects, while the (+)-dobutamine predominantly stimulates the β_1_ and β_2_ adrenoreceptors and exhibits α_1_ antagonism [20,41]. In a dose-dependent manner, dobutamine boosts myocardial contractility and CO due to its effects on the β_1_ adrenergic receptors. The heart rate initially remains unchanged and then increases with higher doses, while the stroke volume only increases at lower doses [20,41,42,43]. It is not uncommon to observe positive chronotropy without an improvement in BP values at higher doses. Its influence on the vascular tone is via weak α_1_ activity and strong β_2_ activity—thus, peripheral vasodilation and a reduction in pulmonary vascular resistance can occur [20,42]. An older study on anesthetized dogs reported a dose-dependent rise in systolic aortic pressure during normovolemia, as well as increases in the systolic, diastolic, and mean aortic pressures during hypovolemia (a 10 mL/kg withdrawal of circulating blood volume). With dose increments from 1 to 10 μg/kg/min, the HR, CO, and pulmonary artery pressure were higher during both volume states [44]. Dobutamine at 10 μg/kg/min resulted in improved CO and myocardial performance with the greatest increase in inotropy seen at this dose in isoflurane-anesthetized normovolemic dogs [45]. In dogs, dobutamine induced minimal changes in BP, but increased the cardiac index, which coincided with significant increases in end-tidal carbon dioxide concentration. However, a decrease in the SVR and afterload was evident, which was deemed to be beneficial for the cardiac workload [46]. Similar findings were identified in another canine study, where dobutamine infusions of 1–8 μg/kg/min administered during isoflurane induced hypotension, which resulted in significant, dose-dependent increases in HR and stroke volume, subsequently improving the cardiac index. The concurrent, dose-dependent reduction in SVR due to β_2_ receptor agonism did not allow the MAP to improve [47]. In an endotoxic shock model in dogs, a dobutamine infusion of 5–10 μg/kg/min consistently increased tissue oxygen delivery and consumption; however, oxygen extraction deteriorated [48]. Our study exhibited mild increments in heart rate, especially at high dobutamine doses, with significant increases in arterial BP, CO, MPAP, PAWP, DO_2_, and VO_2_ and an unchanged OER. However, dobutamine failed to consistently maintain an MAP of >65 mmHg, even at the highest dose of 15 μg/kg/min, which is likely attributed to the simultaneous decrease in SVR.

According to the ‘Surviving Sepsis International Guidelines’, norepinephrine is strongly recommended as the first-line drug for treating vasodilatory shock through its α_1_ and β_1_ adrenergic actions that help to improve BP and CO [49]. In recent times, norepinephrine has been regarded as the most common first-line vasopressor for small animals to treat vasodilatory hypotension in emergency and critical care medicine [50]. Norepinephrine is a sympathomimetic amine derived from tyrosine and is structurally similar to epinephrine, although it lacks a methyl group on its nitrogen atom. This chemical difference is attributed to its primary α_1_, α_2_, and β_1_ adrenergic agonism. When compared to epinephrine, norepinephrine-associated peripheral vasoconstriction and venoconstriction in the venous beds (except the coronary vasculature) are more intense, while the β_1_ activity is milder [20,50]. Increases in heart rate and myocardial contractility may be observed with β_1_ agonism, but baroreceptor-reflex-mediated bradycardia is usually more common in response to α_1_- and α_2_-mediated vasoconstriction [20,43]. Potent vasoconstriction can be concerning, potentially leading to decreased organ blood flow [51]. At 0.1–1.5 μg/kg/min in endotoxemic, anesthetized dogs, norepinephrine increased the MAP, cardiac index, and stroke volume index in a dose-dependent manner, with a lack of effects observed on the SVR and HR [48,52]. Even though the tissue oxygen delivery increased, the oxygen extraction significantly diminished with 0.5–1 μg/kg/min [52]. An improvement in CO was only seen at 0.4 μg/kg/min, but no improvement in the mixed venous oxygen saturation was evident in another canine study [53]. Incremental doses of norepinephrine (0.05–2 μg/kg/min) in a recent canine study reported dose-dependent increases in MAP, CO, and oxygen delivery without insignificant changes in SVR. Stroke volume increased at lower doses, and HR increased at higher doses. No change in oxygen consumption and lactate concentrations was noted during infusion [19]. In our study on hypotensive dogs, we observed some contradictory findings, where norepinephrine did cause a baroreceptor-reflex-mediated decrease in HR, with subsequent improvements in arterial BP, CO, MPAP, PAWP, DO_2_, and VO_2_ and an unchanged OER. Additionally, the percentage increase in CO observed with norepinephrine was much lower than that with dobutamine at T1 and T2.

Arginine vasopressin, also referred to as the antidiuretic hormone, is a peptide hormone produced in the hypothalamus and stored or released from the posterior pituitary gland. The prime triggers for arginine vasopressin release are increased plasma osmolality, hypotension, and hypovolemia. A decrease in the circulating blood volume and blood pressure shifts the osmolality–vasopressin response curve in such a way that normal osmolality is only maintained in the presence of increased arginine vasopressin. A diminished arterial baroreceptor reflex will lead to the disinhibition of this hormone during hypotension, eventually leading to its increased secretion [20,21,54]. Similar to the α _1_, β_1_, and β_2_ adrenergic receptors, vasopressin receptors are also excitatory G protein-coupled receptors. There are five vital vasopressin receptors in the body, i.e., V_1_R, V_2_R, V_3_R, oxytocin receptor, and the P_2_ class of purinoreceptors, which cause cellular effects that are specific to the receptor type. Direct systemic vasoconstriction is the end result of V_1_R stimulation. In vitro studies have suggested that arginine vasopressin may have higher vasoconstricting properties than norepinephrine, phenylephrine, and even angiotensin II. Exogenous or synthetic vasopressin is commonly recommended intravenously during cardiopulmonary resuscitation to aid in the return of spontaneous circulation or to treat vasodilatory and hemorrhagic shock [50,51,54]. Vasopressin is the most common second-choice vasopressor for cats and dogs in intensive care units for the treatment of vasodilatory shock [50]. During experimentally induced hemorrhagic shock in dogs, vasopressin significantly improved the arterial blood pressures and SVR index, with a simultaneous reduction in cardiac index. The pressor effect of vasopressin was steady and lasted longer than epinephrine [55]. In a similar canine model, three doses of vasopressin were compared (0.1 IU/kg, 0.4 IU/kg, and 1.6 IU/kg), and it was observed that the administration of high- and middle-dose vasopressin caused a superior increase in blood pressure and systemic vascular resistance when compared to low-dose. A total of 0.4 IU/kg of vasopressin was the most effective dose for improving hemodynamics during the decompensatory phase of hemorrhagic shock in dogs [56]. Additionally, vasopressin administration before crystalloid resuscitation was observed to be more beneficial in improving the hemodynamic and oxygen delivery functions in dogs with acute hemorrhage [57]. Our study indicated that vasopressin not only failed to stabilize the MAP values to >65 mmHg, but also had a significant negative impact on CO, DO_2_, and VO_2_. These findings warrant caution, as vasopressin infusion, when selected as a therapy for isoflurane hypotension, can compromise CO and thus organ perfusion, causing detrimental effects on patient health.

The need for fluid resuscitation in anesthetized small animals is routinely based on key cardiovascular variables, such as BP. Physiologically supported by the Frank–Starling cardiac curve, a crystalloid fluid bolus can serve as an intervention that is aimed at improving tissue perfusion by increasing a venous return and CO, with a subsequent improvement in BP. However, clinicians must keep in mind that blood pressure is influenced by not just the blood flow, but also the arterial vascular tone. In this regard, the concept of arterial load is significant to understanding the ventricular–arterial coupling [58]. The Windkessel model focuses on the net afterload and the inclusion of net arterial compliance, SVR, characteristic aortic impedance, total arterial inertance, and the effects of arterial wave reflections. Historically, the physiologic basis of arterial load evaluation was based on a two-element Windkessel model of arterial circulation comprising static and dynamic components. The static component describes the pressure–volume relationship and is supported by a resistive component, i.e., SVR, and a pulsatile component, i.e., net arterial compliance. The integrative parameter of arterial load is the effective arterial elastance, which amalgamates both of these components, but was not studied in the present study. Our study dogs displayed decreases in their SVR with the dobutamine and hetastarch treatments and improvements in their SVR values with norepinephrine and with vasopressin more strongly. On the other hand, dobutamine and norepinephrine caused decreases in the PVR, while vasopressin and hetastarch significantly increased this parameter.

Our experimental canine study had multiple limitations. The sample size was small, although adequate for maintaining the statistical power of the study, as per the power analysis. One of the influencing reasons behind this was ethical considerations and restricting unnecessary animal use. This model was based in a research environment; thus, the factors that could potentially influence the hemodynamics were strictly monitored and controlled, such as body temperature, end-tidal carbon dioxide concentration, sympathetic stimulation, and anesthetic depth. Moreover, after achieving a severe hypotension phase where the MAP was <45 mmHg, the vaporizer setting was not changed to lower the ET_ISO_, which is a common clinical intervention and one of the first steps for treating hypotension in small animals. This was crucial for the testing of our study hypothesis and determining which of the four treatments was the most effective in treating isoflurane-induced hypotension, as well as investigating what the effective dosage regimen for each was. We periodically monitored the parameters that could indicate renal perfusion and function, not just during the phases of hypotension, but also while studying the impact of the vasopressor, inotrope, and hetastarch infusions. The small number of timepoints (baseline, T0, T1, and T2) for the data collection restricted the number of paired observations for robust comparisons of the hemodynamic variables between treatments. The treatments had a predetermined dose assigned to them, so it was difficult to predict what the clinical response would have been outside of the dose range, or if more time had been given for the clinical response to occur. Additionally, the researchers were not blinded to the treatments, which could contribute to a bias. However, the study goal was to describe the hemodynamic effects of the treatments during severe hypotension and describe a dosage regimen, which made this scenario inevitable. Lastly, this study utilized healthy Beagle dogs as its study population. Hence, evaluating these treatments and their effects on the hemodynamics in anesthetized canine patients with systemic diseases is warranted.

## 5. Conclusions

In healthy, anesthetized Beagle dogs undergoing acute, severe hypotension (MAP < 45 mmHg) induced by high doses of isoflurane, alongside pharmacologic interventions such as dobutamine, norepinephrine, vasopressin, and hetastarch, norepinephrine administered as an infusion was the most efficient therapy for correcting inhalant-anesthetic-induced hypotension at 0.44 ± 0.19 μg/kg/min. The dobutamine infusion aided in improving CO, DO_2_, and VO_2_, but was unsuccessful in stabilizing the MAP to >65 mmHg even at higher doses, which instead caused a simultaneous reduction in SVR. Vasopressin’s strong vasoconstriction was evident from the elevations observed in SVR; however, it negatively influenced CO, DO_2_, and VO_2_, which could be concerning for both critically ill and healthy canine patients. The hetastarch response to arterial BP was minimal, with a marginal augmentation of CO, DO_2_, and VO_2_, suggesting an inadequate hemodynamic response during the volatile anesthetic-induced vasodilatory state. Further research on canine patients suffering from systemic diseases anesthetized for surgical, medical, or diagnostic procedures is imperative to help to compare these data with the findings of our study. Volatile anesthetic-induced hypotension is one of the most commonly occurring cardiovascular complications in small animals during the intraoperative phase; thus, knowledge regarding the best therapeutic approach to correcting BP could be key for the optimization of patient monitoring and management in anesthetized animal patients.

## Figures and Tables

**Table 1:** Mean ± standard deviation of the measured parameters, i.e., the end-tidal concentration of isoflurane (ET_ISO_), the peripheral oxygen saturation of hemoglobin (SpO_2_), heart rate (HR), systolic arterial pressure (SAP), mean arterial pressure (MAP), diastolic arterial pressure (DAP), intermittent bolus pulmonary artery thermodilution cardiac output (CO), central venous pressure (CVP), mean pulmonary artery pressure (MPAP), and pulmonary artery wedge pressure (PAWP) recorded in ten healthy, mechanically ventilated, isoflurane-anesthetized Beagle dogs (missing data from the other two dogs were eliminated) subjected to four pharmacological interventions used to treat isoflurane-induced hypotension. Baseline data were collected before DOB (DOB_baseline_), NEP (NEP_baseline_), VAS (VAS_baseline_), and hetastarch (HET_baseline_) treatments. Immediately after these treatments, hypotension was induced by increasing the end-tidal isoflurane concentration to achieve MAP < 45 mmHg. After 10 min of maintaining MAP < 45 mmHg, data were collected before starting treatment (i.e., DOB_T0_, NEP_T0_, VAS_T0_, and HET_T0_). The intravenous dosage regimens for the four treatments were: (i) DOB—starting dose of 5 μg/kg/min, which was increased by 50% every five minutes until MAP was 65 to 80 mmHg or max dose of 15 μg/kg/min; (ii) NEP—starting dose of 0.1 μg/kg/min, which was increased by 50% every five minutes until MAP was 65 to 80 mmHg or max dose of 2 μg/kg/min; (iii) VAS—starting dose of 0.5 mU/kg/min, which was increased by 50% every five minutes until MAP was 65 to 80 mmHg or max dose of 5 mU/kg/min; and (iv) HET—6% 670/0.75 hetastarch administered at 1 mL/kg/min until MAP was 65 to 80 mmHg or max dose of 20 mL/kg. Once the treatment was initiated and target MAP was achieved (65 to 80 mmHg), data were collected after 15 min of stabilization (i.e., DOB_T1_, NEP_T1_, VAS_T1_, and HET_T1_) and 15 min after timepoint T1 (i.e., DOB_T2_, NEP_T2_, VAS_T2_, and HET_T2_). There was a minimum 15 min washout period between treatments, where MAP values were ≥65 mmHg.

Treatment Timepoint	ET_ISO_ (%)	SpO_2_ (%)	HR (bpm)	SAP (mmHg)	MAP (mmHg)	DAP (mmHg)	CO (L/min)	CVP (mmHg)	MPAP (mmHg)	PAWP (mmHg)
DOB_baseline_	1.4 ± 0.1	99 ± 1	100 ± 15	118 ± 8	72 ± 9	49 ± 7	1.75 ± 0.32	4.0 ± 0.3	11 ± 3	7 ± 2
NEP_baseline_	1.5 ± 0.1	99 ± 2	96 ± 11	115 ± 7	69 ± 10	46 ± 8	1.69 ± 0.44	3.6 ± 0.2	10 ± 2	6 ± 1
VAS_baseline_	1.5 ± 0.0	98 ± 2	102 ± 12	120 ± 8	68 ± 9	42 ± 8	1.72 ± 0.25	3.8 ± 0.1	11 ± 2	7 ± 2
HET_baseline_	1.5 ± 0.1	98 ± 1	93 ± 10	119 ± 7	71 ± 7	47 ± 6	1.64 ± 0.29	3.4 ± 0.1	11 ± 3	7 ± 1
DOB_T0_	3.0 ± 0.2 *	99 ± 1	107 ± 12	89 ± 5 *	42 ± 4 *	19 ± 4 *	0.88 ± 0.13 *	4.6 ± 0.4	7 ± 1 *	3 ± 1 *
NEP_T0_	2.9 ± 0.1 ^†^	99 ± 1	100 ± 11	86 ± 3 ^†^	41 ± 3 ^†^	18 ± 5 ^†^	0.95 ± 0.19 ^†^	4.1 ± 0.3	6 ± 2 ^†^	4 ± 1 ^†^
VAS_T0_	3.0 ± 0.1 ^‡^	98 ± 1	108 ± 10	90 ± 5 ^‡^	44 ± 2 ^‡^	21 ± 4 ^‡^	0.99 ± 0.11 ^‡^	4.4 ± 0.2	7 ± 2 ^‡^	3 ± 1 ^‡^
HET_T0_	3.0 ± 0.1 ^§^	98 ± 1	99 ± 11	85 ± 5 ^§^	43 ± 2 ^§^	22 ± 4 ^§^	0.87 ± 0.15 ^§^	4.0 ± 0.2	6 ± 2 ^§^	4 ± 1 ^§^
DOB_T1_	3.0 ± 0.1 *	98 ± 2	115 ± 10	123 ± 6	63 ± 3 *	33 ± 4 *	2.10 ± 0.32 *	4.9 ± 0.2 *	11 ± 2	7 ± 1
NEP_T1_	2.9 ± 0.2 ^†^	98 ± 2	76 ± 11 ^†,c^	130 ± 5 ^†,c^	72 ± 6 ^c^	43 ± 3 ^c^	1.26 ± 0.40 ^†,c^	4.6 ± 0.2 ^†^	15 ± 3 ^†,c^	10 ± 2 ^†,c^
VAS_T1_	3.0 ± 0.1 ^‡^	99 ± 2	80 ± 9 ^‡,c^	102 ± 5 ^‡,c^	58 ± 4 ^‡^	36 ± 5 ^‡^	0.76 ± 0.16 ^‡,c^	4.8 ± 0.3 ^‡^	14 ± 3 ^‡,c^	8 ± 2
HET_T1_	3.0 ± 0.1 ^§^	98 ± 1	134 ± 12 ^§,c^	83 ± 5 ^§,c^	47 ± 5 ^§,c^	29 ± 4 ^§^	1.06 ± 0.24 ^§,c^	5.9 ± 0.3 ^§,c^	19 ± 2 ^§,c^	10 ± 2 ^§,c^
DOB_T2_	2.9 ± 0.1 *	98 ± 1	120 ± 11 *	122 ± 7 *	60 ± 6 *	29 ± 5 *	1.97 ± 0.22 *	5.1 ± 0.1 *	13 ± 2	7 ± 1
NEP_T2_	2.9 ± 0.1 ^†^	98 ± 1	70 ± 10 ^†,d^	133 ± 8 ^†,d^	75 ± 8 ^d^	46 ± 6 ^d^	1.40 ± 0.31 ^†,d^	4.8 ± 0.2 ^†^	17 ± 3 ^†,d^	13 ± 2 ^†,d^
VAS_T2_	3.0 ± 0.1 ^‡^	98 ± 2	77 ± 8 ^‡,d^	109 ± 5 ^‡,d^	61 ± 7 ^‡^	37 ± 4 ^‡^	0.68 ± 0.23 ^‡,d^	4.7 ± 0.2 ^‡^	16 ± 2 ^‡,d^	10 ± 2
HET_T2_	3.0 ± 0.1 ^§^	98 ± 1	138 ± 13 ^§,d^	84 ± 6 ^§,d^	48 ± 5 ^§,d^	30 ± 5 ^§^	1.14 ± 0.17 ^§,d^	7.4 ± 0.2 ^§,d^	21 ± 3 ^§,d^	14 ± 1 ^§,d^

* Significant difference (*p* < 0.05) between DOB_baseline_ and DOBT0, DOBT1, and DOBT2; ^†^ significant difference (*p* < 0.05) between NEP_baseline_ and NEPT0, NEPT1, and NEPT2; ^‡^ significant difference (*p* < 0.05) between VAS_baseline_ and VAST0, VAST1, and VAST2; ^§^ significant difference (*p* < 0.05) between HET_baseline_ and HETT0, HETT1, and HETT2. No significant difference (*p* < 0.05) noted between DOB_baseline_ and NEP_baseline_, VAS_baseline_, and HET_baseline_; no significant difference (*p* < 0.05) noted between DOBT0 and NEPT0, VAST0, and HETT0; ^c^ significant difference (*p* < 0.05) between DOBT1 and NEPT1, VAST1, and HETT1; and ^d^ significant difference (*p* < 0.05) between DOBT2 and NEPT2, VAST2, and HETT2.

**Table 2:** Mean ± standard deviation of the calculated parameters, i.e., systemic vascular resistance (SVR), pulmonary vascular resistance (PVR), arterial oxygen content (CaO_2_), venous oxygen content (CvO_2_), oxygen delivery (DO_2_), oxygen consumption (VO_2_), and oxygen extraction ratio (OER) recorded in ten healthy, mechanically ventilated, isoflurane-anesthetized Beagle dogs (missing data from the other two dogs were eliminated) subjected to four pharmacological interventions used to treat isoflurane-induced hypotension. Baseline data were collected before DOB (DOB_baseline_), NEP (NEP_baseline_), VAS (VAS_baseline_), and hetastarch (HET_baseline_) treatments. Immediately after these treatments, hypotension was induced by increasing the end-tidal isoflurane concentration to achieve MAP < 45 mmHg. After 10 min of maintaining MAP < 45 mmHg, data were collected before starting treatment (i.e., DOB_T0_, NEP_T0_, VAS_T0_, and HET_T0_). The intravenous dosage regimens for the four treatments were: (i) DOB—starting dose of 5 μg/kg/min, which was increased by 50% every five minutes until MAP was 65 to 80 mmHg or max dose of 15 μg/kg/min; (ii) NEP—starting dose of 0.1 μg/kg/min, which was increased by 50% every five minutes until MAP was 65 to 80 mmHg or max dose of 2 μg/kg/min; (iii) VAS—starting dose of 0.5 mU/kg/min, which was increased by 50% every five minutes until MAP was 65 to 80 mmHg or max dose of 5 mU/kg/min; and (iv) HET—6% 670/0.75 hetastarch administered at 1 mL/kg/min until MAP was 65 to 80 mmHg or max dose of 20 mL/kg. Once the treatment was initiated and target MAP was achieved (65 to 80 mmHg), data were collected after 15 min of stabilization (i.e., DOB_T1_, NEP_T1_, VAS_T1_, and HET_T1_) and 15 min after timepoint T1 (i.e., DOB_T2_, NEP_T2_, VAS_T2_, and HET_T2_). There was a minimum 15 min washout period between treatments, where MAP values were ≥65 mmHg.

Treatment Timepoint	SVR (dyn · s/cm^5^)	PVR (dyn · s/cm^5^)	DO_2_ (mL/min)	VO_2_ (mL/min)	OER (%)
DOB_baseline_	3108 ± 387	182 ± 14	362 ± 19	77 ± 8	21 ± 2
NEP_baseline_	3095 ± 293	189 ± 15	321 ± 16	71 ± 7	22 ± 2
VAS_baseline_	2986 ± 327	186 ± 12	326 ± 14	79 ± 9	24 ± 1
HET_baseline_	3297 ± 304	195 ± 13	336 ± 17	68 ± 6	20 ± 2
DOB_T0_	3400 ± 319	363 ± 21 *	168 ± 12 *	39 ± 9 *	23 ± 2
NEP_T0_	3107 ± 358	336 ± 20 ^†^	185 ± 15 ^†^	43 ± 7 ^†^	23 ± 3
VAS_T0_	3200 ± 304	323 ± 22 ^‡^	191 ± 13 ^‡^	41 ± 7 ^‡^	21 ± 1
HET_T0_	3586 ± 371	367 ± 19 ^§^	170 ± 9 ^§^	36 ± 6 ^§^	21 ± 2
DOB_T1_	2213 ± 385 *	153 ± 16 *	413 ± 11	90 ± 7	22 ± 1
NEP_T1_	4279 ± 406 ^†,c^	318 ± 14 ^†,c^	244 ± 10 ^†,c^	56 ± 7 ^†,c^	23 ± 1
VAS_T1_	5600 ± 392 ^‡,c^	631 ± 20 ^‡,c^	146 ± 13 ^‡,c^	31 ± 6 ^‡,c^	21 ± 2
HET_T1_	3101 ± 333 ^c^	679 ± 23 ^§,c^	206 ± 9 ^§,c^	47 ± 6 ^§,c^	23 ± 2
DOB_T2_	2067 ± 402 *	244 ± 21 *	386 ± 12	88 ± 6	22 ± 1
NEP_T2_	4011 ± 366 ^†,d^	228 ± 18 ^†^	266 ± 9 ^†,d^	60 ± 5 ^†,d^	23 ± 2
VAS_T2_	6343 ± 459 ^‡,d^	705 ± 22 ^‡,d^	129 ± 11 ^‡,d^	28 ± 5 ^‡,d^	22 ± 1
HET_T2_	2849 ± 427 ^§,d^	491 ± 24 ^§,d^	222 ± 10 ^§,d^	50 ± 7 ^§,d^	23 ± 1

* Significant difference (*p* < 0.05) between DOB_baseline_ and DOB_T0_, DOB_T1_, and DOB_T2_; ^†^ significant difference (*p* < 0.05) between NEP_baseline_ and NEP_T0_, NEP_T1_, and NEP_T2_; ^‡^ significant difference (*p* < 0.05) between VAS_baseline_ and VAS_T0_, VAS_T1_, and VAS_T2_; ^§^ significant difference (*p* < 0.05) between HET_baseline_ and HET_T0_, HET_T1_, and HET_T2_. No significant difference (*p* < 0.05) noted between DOB_baseline_ and NEP_baseline_, VAS_baseline_, and HET_baseline_; No significant difference (*p* < 0.05) noted between DOBT0 and NEPT0, VAST0, and HETT0; ^c^ significant difference (*p* < 0.05) between DOBT1 and NEPT1, VAST1, and HETT1; and ^d^ significant difference (*p* < 0.05) between DOBT2 and NEPT2, VAST2, and HETT2.

## Data Availability

Data supporting the central findings of this research study are contained within the article. Other data pertaining to studying animals may be available on request and are subjected to evaluation on a case-by-case basis respecting the Virginia Polytechnic Institute and State University regulations and policies on data handling.
